# Building political and financial support for science and technology for agriculture

**DOI:** 10.1098/rstb.2012.0274

**Published:** 2014-04-05

**Authors:** Roger N. Beachy

**Affiliations:** 1 Department of Biology, Washington University in St Louis, St Louis, MO 63105, USA; 2 Donald Danforth Plant Science Center, Washington University, One Brookings Drive, St Louis, MO 63132, USA

**Keywords:** agriculture, research support, funding

## Abstract

The high rate of return on investments in research and development in agriculture, estimated at between 20- and 40-fold, provides a strong rationale for increasing financial support for such research. Furthermore, the urgency to provide sufficient nutrition for a growing population amid growing demands for an expanding bioeconomy, while facing population growth and changing global weather patterns heightens the urgency to expand research and development in this field. Unfortunately, support by governments for research has increased at a fraction of the rate of increases in support of research for health, energy, etc. Although there have been significant increases in investments by the private sector over the past two decades, much of the foundational research that supports private-sector activities is generated in the public sector. To achieve the greatest benefits of breakthroughs in research, it may be necessary to reconfigure research funding and technology transfer mechanisms in order to more rapidly apply discoveries to local needs as well as to global challenges. Some changes will likely require significant organizational, administrative and operational changes in education and research institutions.

## 1. Introduction

Among the grand challenges that face society in this century, security in food and nutrition is at or near the top of the list. While some may quibble about whether there are currently more or less than one billion underfed people globally, the numbers are much too high and are exacerbated by weak economies and reduced grain production caused by adverse weather conditions in key production areas. Furthermore, both quantity and quality of nutrition is critical; for example, between 250 000 and 500 000 annually suffer from blindness as a result of deficiencies of vitamin A, and the vulnerable are young children. The high glycaemic index of rice is a cause of high rates of type 2 diabetes in countries where a rice-rich diet is preferred. The vulnerable need more and better foods.

In addition to food production *per se*, the growing focus on agriculture as a supplier of many different products that together support a vigorous and sustainable bioeconomy reminds us of the many challenges that lie ahead, and the importance of a vigorous research environment that will contribute to meeting the challenges. Furthermore, different components of the research and food systems must work in concert to achieve global security in food and nutrition, and it is essential that the components follow a suitable roadmap, including a plan for agriculture, that addresses local, regional or global goals.

A report released in 2009 from the National Research Council of the US National Academy of Sciences entitled ‘A new biology for the 21st century’ [1] identified some of the grand challenges for society: the list includes ensuring health, a safe and sufficient water supply, sustainability and renewable energy supplies. It is notable that agriculture plays an important role in each of these challenges. The New Biology report went on to urge significant investments in multidisciplinary research that engages many different sciences, to address grand challenges. The report made reference to the importance of fundamental research in plant sciences as a component of achieving the grand challenges.

Multiple factors determine whether or not a specific locality, country or global region is or is not secure in accessing sufficient food and nutrition for its population. The factors are generally established by specific government policies, the quality of natural resources, market access and international trade, and education of those that are involved in the many components of the ‘food security’ equation. Of course, food security depends on much more than science and the technologies/practices of agriculture or available natural resources: a key determining factor is the complex set of policies that determine whether or not agriculture is or is not a country priority.

‘Agricultural research’ describes a very broad set of disciplines and each plays a significant role in food security: for example, the science and technologies that lead to practices to prepare the soil, plant and cultivate the crop, and safely store harvested materials are each important. Regardless of the descriptors, chief among the factors that determine the success of agriculture *per se* is research, both fundamental and technical; research has been the primary factor in increasing agricultural yields over the past two centuries.

In the United States, agricultural research became a high national priority with the establishment of the Department of Agriculture and the Land Grant Universities (LGUs) during the presidency of Abraham Lincoln. Research conducted in the land grant and other research universities as well as in private seed and chemical companies during the past 150 years is responsible for the great successes in production, ensuring a safe supply of food and in managing the natural resources of agricultural lands. Not all of the goals for agricultural research have been achieved and not all of the achievements led to sustainable practices but enabled continuing forward progress in crop and food production. The need for continuing investments in fundamental and translational research in public sector institutions remains high and perhaps is more important today than in the past. A similar recommendation was made in a report prepared by the Royal Society [2], in which there was special emphasis on the needs in resource-poor countries.

Unfortunately, the success of the US model for achieving sufficiency of agricultural production is but one part of achieving global food security for a growing population in a climate-vulnerable world. Indeed, the US model is most effective in regions that have a rich natural resource base and a country commitment to agriculture. Fuglie & Schimmelpfennig [3] provide an analysis of the rates of growth in agricultural productivity (all factors considered) in different countries or regions around the globe. As expected, there is a strong correlation between increase in production, good natural resources and government investments in research and development. Brazil, Argentina, India and China are examples of countries where such investments have resulted in high gains in productivity. There are additional countries that have highly productive (or potentially so) agricultural lands that have much slower growth in productivity; these countries are likely to be significant global players in crop production in coming years. Countries in this category include Ukraine, Russia, Indonesia, Mozambique, Tanzania and others. Key to the success in agriculture of any country is that they make significant investments in research, in particular in the plant and animal sciences, and natural resource management that underpin successful, sustainable and productive agriculture.

## 2. Achieving security in food and nutrition requires investments in science, technology and education/outreach

Investments in the sciences that fuelled the growth of agriculture in the USA began in the mid- to late-1800s; it should be noted, however, that agriculture became successful not solely as a consequence of support of agricultural sciences *per se*, but by investments in fundamental and translational science in a variety of disciplines, including in engineering. Following establishment of the LGUs, the federal government has developed programmes to support research at these universities, and in 1914, it developed a formal programme for extension and outreach to transfer technologies to farmers. However, not all of the advances in agriculture came through support of the LGUs, but through support for fundamental and translational sciences by other federal agencies that were established by the mid-twentieth century. This resulted in extraordinary discoveries but not all were immediately relevant to agriculture; some examples include

— In 1914, development of hybrid maize was reported; yet, it was not until mid-century that hybrid seeds became widely available and accepted by farmers and consumers.
— The use of non-organic fertilizers began in the nineteenth century, but it was not until the mid-twentieth century that discoveries in chemistry led to development of the Haber–Bosch process to efficiently develop chemically fixed nitrogen; its use in agriculture became widely accepted.
— The report of ‘jumping genes’ in maize in 1931 derived from basic research, made possible by research in genetics using other organisms, led to accurate genetic maps for maize that facilitated the integration of knowledge gained by DNA sequencing in the twenty-first century to methods used by modern maize breeders.
— The National Science Foundation (NSF) and the National Institutes of Health (NIH) were established in 1950 and 1958, respectively, flanking the report of the structure of DNA in 1953.
— The Agriculture Research Service (ARS), the intramural research agency of the US Department of Agriculture (USDA) was established in the mid-1950s.
— By the late 1940s and onwards, the widespread use of fundamental discoveries in genetics were being applied to improvement of many different crops, and by the early 1960s, the heralded Green Revolution was underway.
— Advances made in genetics, largely founded on the structure of DNA, discovery of its transcription to RNA, and its translation to proteins were made possible by research in plant, animal and bacterial viruses and model organisms funded by the NSF and the NIH.
— In 1972, the first reports of laboratory studies in recombinant DNA were made, a discovery that would lead to a revolution in applied biology that led to advances in health sciences and in agriculture.
— From the 1970s, at approximately decadal intervals, studies by working groups inside and outside of Congress, including studies by the National Research Council/National Academy of Sciences urged greater support for fundamental research in the sciences that impact agriculture: interagency working groups were established, and a report on applied genetics was published by the Office of Technology Assessment. A report from the Winrock Foundation urged that agricultural research be made competitive and open to all scientists rather than only to those in schools of agriculture.
— By the early 1980s, fundamental discoveries made decades earlier led to development of the first genetically engineered (transgenic) plants.
— Applications of discoveries based on DNA sequence and rDNA technologies led to marker-assisted selection (MAS) of traits by plant breeders.
— By the year 2000, DNA sequencing was completed on a single strain of *Arabidopsis thaliana*, a model plant.
— By the end of the last decade, DNA sequencing technologies had revolutionized studies of many different species, and today proposals are being made to sequence up to 10 000 land races of rice as a way to discover and use the vast biodiversity of its genome; studies of the soil microbiome that contribute to plant productivity got underway.
— Recent advances in climate sciences remind us that accurate predictions of long-term weather patterns remain elusive but will help plant scientists execute efforts to develop crops that are more resilient to changes in climate.
— Advances in information technologies, and bioinformatics in particular, is adding great value to plant and agricultural scientists who collate vast databases from DNA sequencing, crop phenotyping, global mapping of weather patterns, soil conditions and agricultural production, changing consumer patterns, etc., as they align their research to most effectively serve society.

These cursory comments of past investments in diverse research disciplines that have benefited agriculture and food security are intended to make a single point: investment in research in a multiple of disciplines is important for the success of agriculture and the food supply.

Many countries have historically lacked the wealth to invest in the range of research supported by wealthy nations: it follows that lack of support for agriculture is in part responsible for the food and nutrition insecurity in many countries. Fortunately, the growing economies of China, Brazil and India are making significant investments in a range of sciences much of which directly or indirectly impacts agriculture. It is not a surprise, then, that countries in which agriculture is a sizeable component of the economy make investments in research that support it. Table 1 presents a summary of the impact of public investments in research and development (R&D) in agriculture on the contributions to gross domestic product (GDP), and show significant returns on investment for different crops. Furthermore, increases in crop production can reduce poverty in rural areas, including of smallholder farmers as well as of large-scale farming. Data such as these should influence policy- and decision-makers in the countries that strive to achieve food and agricultural security to increase commitments to increasing internal research.

**Table 1.** Dollar returns, in terms of GDP or agricultural GDP, owing to a dollar increase in public investment in agricultural commodities, 2006–2010. Adapted from [4]. (Table 4.2 in [5].)

subsector	GDP	ag. GDP
cereal grains	2.75	2.73
maize	7.02	6.59
paddy rice	1.41	1.22
wheat	5.34	5.15
roots and tubers	5.03	4.65
cassava	5.46	4.61
Irish potatoes	5.88	5.66
sweet potatoes	2.53	2.22
livestock	2.02	1.90
poultry	10.54	10.09
other	1.81	1.74
pulses	9.09	8.21
fishery	12.50	12.35
cash and export crops	1.02	1.24
coffee	1.01	1.74
tea	1.95	2.52
bananas	5.35	4.94
oilseeds	5.89	4.73
other	1.08	1.07
staple crops and livestock	3.84	3.63
agriculture, total	3.19	3.11

## 3. Multidisciplinary research yields high pay-off in agriculture

Research outcomes in knowledge-intensive enterprises such as agriculture are the result of prior investments made 10–15 years earlier; recent advances in science and technology have been exciting both in discovery of new knowledge and by contribution to very rapid development of crop varieties that address needs in agriculture. For example,

— *Plant breeding.* Advances in DNA sequencing technologies have made possible determination of the genomes of many crops, including maize and other cereals, as well as many different vegetables and their wild relatives. In general, sequencing priorities are established based on scientific interest, potential for commercial impact and available funding. Knowledge of sequences is used to increase the rate of classical breeding through use of DNA sequence ‘markers’ known to confer specific traits. Such knowledge is also used to isolate specific gene sequences for basic research and for crop genetic engineering.
— *Plant production and protection.* Research to better understand how crop and non-crop plants respond to environmental stresses is used to develop crops that are durably resistant to stresses. By combining forward and reverse genetic approaches with studies in physiology and agronomy, researchers are characterizing genes and metabolic processes that are negatively or positively impacted by environmental conditions. This has led to experiments that introduce genes that may confer heat or drought tolerance to plants that are susceptible to such stresses. Similarly, genes that confer resistances to fungi, bacteria, viruses and nematodes have been identified and tested in susceptible plant varieties, some of which confer tolerance or immunity to one or more diseases. With greater knowledge, it will be possible to develop crop varieties with durable, long-lasting resistance to multiple pathogens and pathotypes. Other studies target identification of genes that enhance the uptake and use of fixed nitrogen, phosphorus, potassium and other essential nutrients more effectively, so that farmers can use lower amounts of fertilizers and thus reduce negative effects of overuse.
— *Genetic engineering.* Introducing genes from different plant and bacterial sources via ‘transgenic’ approaches makes it possible to add new traits to plants and other organisms and significantly expand the pool of genetic diversity that can be used in agriculture. Newer methods, including site-directed mutagenesis, and targeted integration of transgenes to specific sites in the genome, and use of artificial chromosomes further expand options for plant breeders.
— *Sustainability of agricultural practices.* Modern genomic sciences and improved tools for analysing data are proving to be invaluable to studies to achieve sustainable agriculture. For example, studies of the role of soil microbiomes in soil fertility and of microbes in plant diseases and disease prevention will contribute to sustainable agriculture. Some of the microbes are valued for contributions to plant nutrition; others provide biotic protection against pests and disease-causing organisms and will reduce the need for chemical protectants. Newer studies in ‘ionomics’ define the role of the mineral content of soils to crops productivity under environmental stresses. Such research will lead to agriculture that ‘nurtures’ the soil to maximize crop productivity; it may also lead to practices that match the chemical and biological profiles of soils with the genetics of the seed and to optimize soil moisture to benefit both microbes and plants.
— *Developing a viable bioeconomy.* Plants produce a wealth of different types of chemical compounds, estimated by ecologists and chemists to be more that 400 000 across the diversity of plant species. Identifying the role of these constituents in plant growth and metabolism, including in innate mechanisms of resistance to disease are active areas for research. Plant constituents also contribute to nutrition, applications in pharmaceutical and industrial chemistry, bioenergy, biomaterials, among other uses. As the pathways of metabolism are more fully elucidated, scientists apply tools of metabolic engineering of plants and microbes to develop products that have value in these and other fields.

## 4. Investment in research leads to more successful agriculture

**Figure 1.**
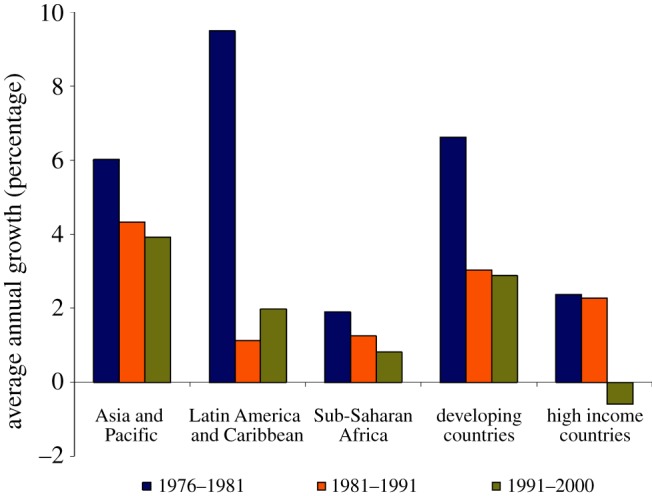
Countries around the globe have, in general, decreased the growth in support for agricultural research over the last three decades; with the exception of countries in Latin America. The significant decline in annual investment in high income countries between 1991–2000 is especially troubling [6]. (Online version in colour.)

Unfortunately, countries that decreased their investments in R&D in agriculture over the past decades are not positioned to benefit from recent advances in knowledge and technologies. Figure 1 presents a glimpse of changes in the rate of growth in funding for agricultural research over the past four decades, a period during which wealthy countries (i.e. rich in both financial and natural resources) produced an overabundance of agricultural products: memories of surplus grain, sugar, milk and other commodities remain in the minds of many. Abundance followed strong investment in public research that, on a GDP basis, was significantly greater years ago than it is today; in many countries with high incomes, there has been significant reduction in such investments. The recent reports of increased funding for agricultural research in China, Brazil and India are an indication of the importance of agriculture to these countries [6]. China, in particular, has greatly increased funding for fundamental plant and agricultural sciences, including establishing key laboratories led by outstanding scientists and innovators. As a consequence, there has been a dramatic increase in high-quality scientific publications from China. This is certainly a positive sign for agriculture of the future.

Over the last decade, a small number of African nations have committed to increasing investments in agricultural research, although most of the funding is directed to applied research rather than directed to fundamental science. This has resulted in increased funding for in country R&D by outside donor countries and foundations for collaborative and internal research, resulting in increased productivity. Hopefully, this will lead to further internal funding for fundamental as well as translational research in the future.

In contrast to support by governments, there has been a steady increase in funding of global R&D in agriculture and food by the private sector in the past 20 years [7] with investments in some countries reaching more that 60% of the total. Of this investment, approximately half has been in the food industry, with lesser investments in animal nutrition and animal health. Of the remainder, the largest share has been to improve seeds of the major commodity crops. Investments in biomass crops for biofuels and other biomaterials are also on the rise; the amount of investment in horticultural crops is less clear but is significantly lower for commodity crops. It is not clear whether increased expenditures in seed technologies has had a significant negative or positive effect on investments in research in agrichemicals, though in the long run research in plant genetics is expected to reduce the use of agrichemicals as a whole.

Much of the success of seed companies during the past two decades relied heavily on fundamental research in plant sciences, and the ‘omics’ sciences that improve the rate and efficiency of modern plant breeding. During the 1980s and early 1990s, several large companies (in particular, Monsanto Company and Dupont/Pioneer Company) invested heavily in internal research and/or in collaborative research with university researchers to support fundamental discovery in plant biology. This stimulated an exciting time in plant biology and attracted a wide range of talent to the field. The Rockefeller Foundation and others extended this excitement to fundamental studies in rice as a model system for small grain crops. In the mid- to late-1990s, the companies began to shift their investments from discovery research to product development and reduced investments in collaborations with academic researchers. At present, companies rely to a greater degree on publically funded research for discoveries that will lead to future products than they did in the 1980s. Unfortunately, there has not been a concomitant increase in public sector funding for research, which leads to the concern of Pardey *et al*. [8] that slowdown in the annual rate of increase in crop production is in large part a consequence of reduced investment by public sector sources. This is troublesome as it comes at a time of increased global demands for agricultural products and concern for impacts of climate change on productivity.

## 5. Building support for plant science and agriculture

As noted earlier, there is a high rate of return on investments in agriculture and in research, and scientists and economists alike are puzzled by the apparent unwillingness of governments in wealthy countries to increase investments given the economic value of agriculture. It is axiomatic that investments made in wealthy countries also lead to pay-offs in less wealthy countries by ‘spill-over effects’ [9]. And, we, in wealthy countries, gain satisfaction in knowing that our efforts impact agriculture in other countries. Nevertheless, research support for food and agriculture is generally not of highest priority in wealthy nations where support of research in other areas, in particular in defence, the health sector, energy and other sciences takes a far greater share of public research dollars.

During my tenure as first Director of the National Institute of Food and Agriculture (which provides funds for extramural research and education/outreach) at the USDA, I participated in discussions to set priorities for research and for aspirational budgets that would fund it. In the USDA, which in this decade, has enjoyed a budget that exceeded $140 billion annually, less than 2% is devoted to intramural and extramural research programmes. The remainder of the budget is directed to social programmes (food assistance) and farm support, rural development, food safety and inspection, plant and animal inspection, regulations in GM agriculture and other programmes. Thus, there are many competing interests for resources, and it is difficult to gain substantial traction to increase the percentage that goes forward as a recommendation to the Administration. In recent years, the outcome has been that significantly less funding is allocated by Congress for agricultural research than recommended by the Administration. The challenges, however, are certainly more difficult in the context of the economic conditions of this decade than in the 1970s and 1980s.

The following reasons for limiting public funding for research in the US (and elsewhere?) are not uncommon:

— *There should be greater reliance on the private sector for research support*: the logic is that if it is worth doing, then certainly the private sector will do it or support research technologies that will enable them to be more successful.
  While there is substantial investment by the private sector in research and product development, much of the fundamental knowledge in this sector is conducted in the public sector. Furthermore, private-sector investments are generally made in crops with large acreage and high seed volume and which provide strong returns on investment. Many important crops with lower profit margins suffer from lack of investment. Without such investment, productivity will lag behind the needs.
— *Advocacy for increasing support for agriculture is weak in the taxpaying community: subsidies for farmers are generally more important to farmers than is research.* Unlike the high degree of public support for biomedical research, defence and other sectors, advocacy for support of food and agriculture is fragmented and therefore weak in impact. Advocacy on behalf of agricultural research has historically been by a variety of special interests rather than on behalf of research *per se*: for example, in support of specific commodities or crops; of methods of production; of food manufacturing; of local economic interests; in support of specific disciplines; in support of translational research and extension, among other interests.
  An additional challenge is that the general urban public lacks awareness of how fundamental and translational research in food and agriculture serves them, if at all. Some consumer groups focus on important issues such as the importance of a sustainable ago-ecosystem, air and water quality associated with agriculture and other issues, often in absence of knowledge of agriculture *per se*. Others focus on accessibility, nutrition and safety of foods.
  Building a broad-based consortium to advocate on behalf of increased support for agricultural research may be essential to gaining the backing of policy-makers who allocate funds. A similar approach has been used to garner support for biomedical research in the US with positive outcomes.
— *Lack of a national plan/roadmap for agriculture and of the role of agriculture in the national economy* can lead to the appearance of lack of relevance of research, duplication of efforts and can dissuade policy-makers from increasing funding. Furthermore, support for research will be enhanced by knowing that there is a ‘master plan’ that serves national or regional goals. Building an impactful roadmap will necessarily involve government officials and policy-makers, as well as leaders of relevant industries, academies of education and research, and may engage the consumer among other stakeholders.
— *Lack of flexibility of the academic research community to collaborate with government and private sector* to achieve specific goals can temper enthusiasm for increasing public support for agricultural research and education. A common theme voiced by private-sector companies is that public-sector researchers are not willing to work in close collaborations with the private sector; and that many graduates are not well trained to work in the private sector. Addressing research interests as well as concerns can require close working relationships between institutions and the private companies sector, and will take adjustments by each partner. We are reminded that engineers have been working closely with industry partners for many years: perhaps, it is timely for closer relationships within the biological sciences.
  A well-described roadmap for agriculture can be an important guide for research institutions, in particular for how new faculty are selected. Research universities usually select faculty that can successfully compete for research grants: if funds are available for agricultural research, then there is greater likelihood that faculty will be hired than if funds are more limited. And, because an effective roadmap by its nature is subjected to regular review and modification faculty interests need to be sufficiently flexible to adapt to changing priorities. While current institutional structures and administrative limitations can limit their flexibility, it will be necessary to make changes that make it possible to adapt to changing priorities.
— *A poorly designated agricultural administrative unit* or a unit that is subtended to another administrative area (for example, in a ministry of environment) rather than to agriculture *per se*, may be poorly supported in budget discussions. In countries where research is separated from agriculture and/or from agricultural education and outreach, it can be difficult to galvanize consistent support for a research agenda for agriculture. Under these circumstances, it will be important to focus on the importance of outcomes to goals set forth in a roadmap to justify support for research.

While there is currently a resurgent interest in agriculture, it is not yet evident that increased funding will be available in the near future.

## 6. Concluding remarks

There is ample evidence to document that investments made in fundamental and translational research leads to increased agricultural productivity. Furthermore, research in allied fields, including in other biological sciences and engineering sciences, can also have ‘knock on’ positive impacts in agriculture. Nevertheless, funding to support these efforts has decreased in real terms over the past two decades, at a time when there are ever-greater demands for products derived from agriculture. As demands of a growing population and an expanding bioeconomy place greater expectation on agriculture and agro-ecosystems, it is critically important that commitments for increased funding be made, country by country, and on a global scale. A roadmap for agriculture will increase the likelihood for greater funding, but may require adjusting administrative structures of research institutions to accommodate increased multidisciplinary activities. Increased funding for these activities will likely include partnerships between foundations, government and private sector. While these and related changes will not be easy, they are part of the future that leads to a stronger agriculture that meets the needs of a growing population in a changing world.
